# Motivations, barriers, and communication recommendations for promoting face coverings during the COVID-19 pandemic: Survey findings from a diverse sample

**DOI:** 10.1371/journal.pone.0251169

**Published:** 2021-05-07

**Authors:** Rhyan N. Vereen, Allison J. Lazard, Simone C. Frank, Marlyn Pulido, Ana Paula C. Richter, Isabella C. A. Higgins, Victoria S. Shelus, Sara M. Vandegrift, Marissa G. Hall, Kurt M. Ribisl

**Affiliations:** 1 Hussman School of Journalism and Media, University of North Carolina, Chapel Hill, North Carolina, United States of America; 2 Lineberger Comprehensive Cancer Center, University of North Carolina, Chapel Hill, North Carolina, United States of America; 3 Gillings School of Global Public Health, University of North Carolina, Chapel Hill, North Carolina, United States of America; 4 Carolina Population Center, University of North Carolina, Chapel Hill, North Carolina, United States of America; Middlesex University, UNITED KINGDOM

## Abstract

State and local health departments have been tasked with promoting the use of face coverings to decrease the spread of COVID-19 in their respective communities. However, little is known about motivations and barriers to wearing face coverings in the context of COVID-19 prevention, particularly among communities of color who are at an increased risk of serious illness from the disease. The purpose of this study was to identify common motivations and barriers to face covering use, as well as explore perceptions of messages encouraging the use of face coverings among a racially and ethnically diverse sample. A survey was distributed electronically to North Carolina (NC) residents through NC Department of Health and Human Services listservs in July 2020. Participants self-categorized as Latino/a (33.5%), Black (39.1%), or white or another race (27.5%). The most commonly endorsed motivations for wearing face coverings were to avoid spreading COVID-19 (77%), as well as to protect people who are vulnerable (76%) and one’s community (72%). Being uncomfortable (40%) was the most commonly endorsed barrier. Messages that included a clear request (ex. please wear a face covering) and a direct benefit (ex. keep community safe) were more commonly endorsed than those that did not. Commonly endorsed motivations, behaviors, and messages differed by race and ethnicity. Increased attention to message content, message structure, and access to information and resources may aid local officials in increasing consistent use of face coverings.

## Introduction

As of late October 2020, over nine million confirmed cases and 229,000 deaths in the United States (US) were attributed to the novel coronavirus disease 2019 (COVID-19) pandemic. The pandemic continues to wreak havoc across the US, but poses a unique threat to Black, Indigenous, and People of Color (BIPOC) who have experienced disparities in COVID-19 diagnoses and mortality. For example, North Carolina (NC) has observed over 274,000 cases and 4,300 deaths from COVID-19 [1]. While Latino/a residents comprise roughly 10% of NC’s population, they have accounted for 36% of the cases in the state. Additionally, Black residents comprise 22% of NC’s population but have accounted for 31% of deaths from COVID-19 in the state [1, 2]. The disparities observed among these populations are driven by a complex set of social determinants of health, which include higher rates of job-related exposure to the SARS-CoV-2 virus and greater barriers to appropriate and timely healthcare access and utilization [3, 4]. Given the challenges of addressing such systemic inequities during the COVID-19 pandemic, nonpharmaceutical interventions to slow the transmission of COVID-19 have instead focused primarily on behavior change campaigns. While communication and behavioral interventions cannot completely prevent the spread of COVID-19, nor eliminate health disparities, they can play a significant role in slowing COVID-19 transmission and improving health outcomes among diverse communities.

### Preventive behaviors

Knowledge about COVID-19 transmission, as well as the efficacy of preventive behaviors, have changed over time. Behaviors that were strongly recommended in the early months of the pandemic (March-May 2020) such as frequent hand washing and social distancing are still encouraged, though public health experts now cite the use of face coverings as the most important behavioral intervention to slow the spread of COVID-19 [5, 6]. Specifically, researchers predict that use of face coverings by at least 80% of the population could slow COVID-19 transmission more quickly and efficiently than a lockdown [7]. At a glance, this finding seems promising given that recent national polls suggest that a majority (76–83%) of Americans wear face coverings in public at least some of the time [8, 9]. However, fewer (44%; Brenan, 2020) wear them all of the time in public, as recommended by the Centers for Disease Control and Prevention (CDC), White House Coronavirus Task Force, and other public health officials [10]. Additionally, findings from national studies assessing the use of face coverings among populations experiencing disparities have been mixed, but suggest that rates of face covering use among African Americans and Hispanic populations may be similar or possibly higher than white populations [9, 11]. Despite seemingly similar patterns of use, lack of consistent face covering use may have a more detrimental effect among BIPOC, who are more likely to be exposed to SARS-CoV-2 and have comorbid conditions that affect COVID-19 outcomes. Ongoing research is needed to determine state-level patterns of face covering use and identify methods to encourage consistent use.

### Communication campaigns

Although the federal government now recommends that individuals wear face coverings in public settings, little guidance has been provided to local officials about how to promote their use. To encourage individuals to comply with public health recommendations, local health departments across the country have created various communication campaigns to encourage face covering use. Communication efforts help the public decipher between scientific recommendations and COVID-19-related misinformation. Therefore, there is a need to gain understanding about messages characteristics that may aid in persuading individuals to partake in recommended COVID-19 protective behaviors [12]. However, there is limited research available to inform the design and implementation of COVID-19-specific campaigns, as at the time of the current study, few studies had examined the public’s attitudes towards face coverings or communication strategies to promote their use during the COVID-19 pandemic [9, 13]. Despite the lack of direct evidence informing current COVID-19 behavioral interventions, existing behavioral frameworks can be used to inform research related to communication campaigns to promote adherence to public health guidelines.

For example, theories of health behaviors highlight constructs of perceived benefits and barriers as key predictors of behavior [14, 15], and findings from several recent studies support the influence of perceived benefits and/or barriers on engagement with COVID-19 prevention behaviors including social distancing and contact tracing activities [16]. Behavioral frameworks may prove especially useful for exploring individuals’ decisions to use or not use face coverings. Identifying motivations and barriers to using face coverings can inform local officials of the communication needs of their community members to aid in increased consistent use of face coverings and mitigating the transmission of COVID-19. The purpose of this study was to identify common motivations for and barriers to face covering use, in addition to exploring perceptions of messages intended to promote the use of face coverings.

## Methods

### Procedures and participants

A screener and survey were distributed electronically through NC Department of Health and Human Services (NC DHHS) listservs in July 2020. Eligible participants reported being at least 18 years old and currently living in NC. The sampling approach was not implemented to achieve a representative sample of NC residents, but to gain insight from a demographically diverse sample, containing individuals from populations facing COVID-19 disparities. Given noted disparities in COVID-19 illness among communities of color [4, 17], recruitment messages were shared across targeted Latino/a/x and Black listserv networks. Due to emerging concerns about increasing incidence of COVID-19 in rural areas and among young adults during the time of recruitment, non-Latino/a white individuals were also eligible if they reported living in a rural area or were between the ages of 18–25 years old. Participants reported whether they were Latino/a (i.e., having Latino, Hispanic, or Spanish origin, regardless of race), Black (i.e., reported being non-Latino/a Black or African American) or white or another race or ethnicity (i.e., not Latino/a or Black). After providing online written informed consent, participants completed an online survey containing items about face covering use, motivations, barriers, and face covering messages. This study was approved by The University of North Carolina Office of Human Research Ethics/Institutional Review Board. Study materials and data are publicly available on the Carolina Digital Repository [18].

### Measures

Face covering use was assessed using the item, “In the past month, how often have you worn a face covering in public when you were unable to stay 6 feet away from other people?” Response options were “never”, “some of the time”, “most of the time”, or “all of the time”.

The survey then included statements to identify reasons participants *want to* (motivations) and do *not want to* (barriers) use face coverings. Statements were rooted in Health Belief Model constructs of perceived benefit, susceptibility, severity, barriers, and self-efficacy [14, 19]. Participants were asked “How much do these reasons make you *want to* wear a face covering?” followed by a randomized list of 18 statements informed by qualitative work (see Table 2 for a complete list of the statements) [20]. Example statements included “Wearing a face covering could prevent me from getting COVID-19” and “I want to protect people who are vulnerable”. Participants were then asked, “How much do these reasons make you *not want to* wear a face covering?” followed by a randomized list of 29 different statements (see Table 3 for a complete list of the statements). Barrier statements included items adapted from a study by Hung [21], as well as items created to assess additional barriers identified in qualitative work (ex. myths and misconceptions about face covering use) [18]. Example barriers included “I don’t know when to wear a face covering” and “Wearing a face covering is a sign of weakness”. Responses for motivational and barrier statements were collected on a 5-point scale ranging from not at all (1) to a great deal (5). Response options were dichotomized to reflect the percentage of participants who positively endorsed the statement “quite a bit” or “a great deal” compared to those who did not (responses “not at all”, “a little”, or “somewhat”). The percentage of participants who endorsed the statements were ordered in descending order to reflect the statements most commonly endorsed among the total sample and by racial and ethnic subgroups.

Participants were then asked “How much do these messages make you *want to* wear a face covering?” and shown a randomized list of 23 messages encouraging the use of face coverings (see Table 4 for a complete list of the messages). The diverse pool of messages were adapted from recent literature on COVID-19-related communication [22], as well as qualitative work [18]. Example messages were “Help keep loved ones safe. Wear a face mask in public.” and “Mask on. To carry on.” Similar to motivational and barrier statements, responses were collected on a 5-point scale ranging from not at all (1) to a great deal (5) and dichotomized to represent the percentage of participants who endorsed the message as one that would make them want to wear a face covering “quite a bit” or “a great deal”, as opposed to “not at all”, “a little”, or “somewhat”. The percentage of participants endorsing the messages were ordered in descending order to reflect the most frequently endorsed messages.

Demographic characteristics included self-reported age (continuous), sex (male, female, or other gender identity), and education (categorized as high school graduate or less, some college or technical school, Associate’s degree, or Bachelor’s degree or more). Participants were also asked whether they have had COVID-19 (categorized as definitely/probably not, not sure, or definitely/probably yes).

### Statistical analyses

Participant characteristics are presented. Descriptive statistics (frequencies and percentages) were generated to assess the prevalence of endorsement of motivations and barriers. The percentages of participants who endorsed the statements are presented. Additional descriptive statistics (mean, standard deviations, frequencies, and percentages) were generated to determine the distributions of response options and frequency of endorsement for each message. The statistical significance of comparisons between these statistics was not of interest in the current study, as the purpose of the current analysis was not to make comparisons between racial and ethnic groups, but to identify commonly endorsed motivations, barriers, and message types among a racially and ethnically diverse sample, as well as among the referenced subgroups. All analyses were conducted in SAS 9.4.

## Results

A total of 448 participants completed the survey (Table 1). Participants identified as Latino/a (33.5%), non-Latino/a Black (39.1%), or non-Latino/a white or another race (27.5%). On average, participants were 33.5 years old (SD: 11.98) and about half (48%) were male. The majority of participants had a college degree (Associate’s degree, 21%; Bachelor’s degree, 46%) and over one-third (38.2%) of participants reported being from a rural area of the state. Few (6%) reported having had COVID-19.

**Table 1:** Characteristics of survey participants by race and ethnicity.

	Total, N = 448	Latino/a, n = 150	Black, n = 175	White or other, n = 123
Characteristics	n (%)^a^			
Age, Mean (SD)	33.5 (11.98)	32.6 (9.33)	32.5 (11.2)	35.9 (15.3)
Age				
18–25	121 (27.0)	23 (15.3)	45 (25.7)	53 (43.1)
26 or older	327 (73.0)	127 (84.7)	130 (74.3)	70 (56.9)
Sex				
Male	215 (48.0)	81 (54.0)	100 (57.1)	34 (27.6)
Female	232 (51.8)	69 (46.0)	74 (42.3)	89 (72.4)
Other or prefer not to answer	1 (0.2)	0 (0.0)	1 (0.6)	0 (0.0)
Education				
High school graduate/GED or less than high school	61 (13.6)	32 (21.3)	18 (10.3)	11 (8.9)
Some college or technical school	89 (19.9)	35 (23.3)	25 (14.3)	29 (23.6)
Associate’s degree	94 (21.0)	36 (24.0)	32 (18.3)	26 (21.1)
Bachelor’s degree or higher	204 (45.5)	47 (31.3)	100 (57.1)	57 (46.3)
Residential area				
Urban	166 (37.1)	75 (50.0)	77 (44.0)	14 (11.4)
Suburban	111 (24.8)	40 (26.7)	45 (25.7)	26 (21.1)
Rural	171 (38.2)	35 (23.3)	53 (30.3)	83 (67.5)
Had COVID-19				
Definitely not or probably not	376 (93.9)	118 (78.7)	159 (90.9)	99 (80.5)
Not sure	46 (10.3)	17 (11.3)	11 (6.3)	18 (14.6)
Definitely yes or probably yes	26 (5.8)	15 (10.0)	5 (2.9)	6 (4.9)
Face covering use				
Never	62 (13.8)	53 (35.3)	6 (3.4)	3 (2.4)
Some of the time	101 (22.5)	38 (25.3)	38 (21.7)	25 (20.3)
Most of the time	168 (37.5)	38 (25.3)	86 (49.1)	44 (35.8)
All of the time	117 (26.1)	21 (14.0)	45 (25.7)	51 (41.5)

^a^Percentages may not total 100% due to missing values.

### Face covering use

Participants reported wearing face coverings in public “never” (13.8%), “some of the time” (22.5%), “most of the time” (37.5%), or “all of the time” (26.1%) (Table 2). Notably, 35% of Latino/a participants reported “never” using a face covering, compared to 3% of Black and 2% of white or other race participants.

**Table 2:** Frequency of endorsement of motivations for face covering use.

	Total, N = 448		Latino/a, n = 150		Black, n = 175		White or other, n = 123	
Statement	%	Order	%	Order	%	Order	%	Order
I don’t want to give COVID-19 to anyone.	77%	**1**	69%	**2**	79%	**3**	84%	**1**
I want to protect people who are vulnerable.	76%	**2**	71%	**1**	77%	5	83%	**2**
Wearing a face covering will prevent my community (e.g., members from my neighborhood, work, university, church) from being at risk.	72%	**3**	68%	**3**	81%	**1**	66%	**4**
I feel a responsibility to wear a face covering.	72%	**4**	65%	**4**	79%	**3**	67%	**3**
Wearing a face covering could prevent me from giving COVID-19 to my family.	69%	**5**	63%	**5**	78%	4	63%	**5**
The sooner we all wear face coverings, the sooner we will be able to “get back to normal”.	66%	6	60%	6	80%	**2**	61%	6
Wearing a face covering helps keep our hospitals from getting too full.	64%	7	57%	7	78%	4	61%	6
I want to follow the experts’ advice.	62%	8	55%	8	75%	6	61%	6
Wearing a face covering could prevent me from getting COVID-19.	59%	9	52%	9	78%	4	53%	8
Wearing a face covering is something I can control in an uncontrollable situation.	57%	10	49%	10	81%	**1**	53%	8
Wearing a face covering will allow NC to ease restrictions.	55%	11	46%	11	71%	7	55%	7
Wearing a face covering will allow schools to open.	52%	12	44%	12	65%	8	46%	9
Wearing a face covering allows me to see my friends and family.	50%	13	41%	13	64%	9	39%	10
I would be embarrassed if people saw me not wearing a face covering.	48%	14	38%	14	56%	10	26%	11
I am afraid of getting in trouble if I don’t wear a face covering.	45%	15	36%	15	52%	11	17%	13
It seems like everyone else is wearing a face covering	43%	16	33%	16	49%	12	20%	12
I feel pressure from friends and family.	41%	17	30%	17	49%	12	20%	12
Wearing a face covering allows me to show off my style.	38%	18	28%	18	36%	13	12%	14

% = Percentage of participants who responded, “quite a bit” or “a great deal” (vs. “not at all,” “a little” or “somewhat”).

Bold numbers denote the 5 most prevalently endorsed statements among the total sample and by race and ethnicity.

### Motivations

In general, participants were most motivated to wear face coverings to protect themselves and loved ones from COVID-19 (Table 2). Specifically, the most commonly endorsed motivations were “I don’t want to give COVID-19 to anyone” (77%) and “I want to protect people who are vulnerable” (76%). Black participants, however, were also motivated by a desire for control and normalcy, with common endorsement of “Wearing a face covering is something I can control in an uncontrollable situation” (81%) and “The sooner we all wear face coverings, the sooner we will be able to get back to normal” (80%).

### Barriers

Being uncomfortable was the most commonly endorsed barrier among the total sample (40%), as well as each subgroup (45% Latino/a, 42% Black, and 30% white or other; Table 3). Other commonly endorsed barriers among Latino/a participants included that “We need to save masks for essential workers” (37%), “Wearing a face covering makes other people think that I am sick” (37%), and “I don’t want to breathe in my own carbon dioxide or germs” (37%). Similarly to Latino/a participants, Black participants commonly endorsed the statement that “We need to save masks for essential workers” (35%), but also endorsed that “I sometimes forget to bring my face covering with me when I leave my home” (37%), “I’m not afraid of getting sick from COVID-19” (37%), and “I don’t know where to find a face covering” (34%). White and other race participants most commonly endorsed statements that “Wearing a face covering makes it difficult for other people to see my facial expressions” (26%), “I don’t need to wear a face covering when I am only around family or friends” (24%), and “I don’t believe wearing a face covering will protect me from getting COVID-19” (24%).

**Table 3:** Barriers to face covering use.

	Total, N = 448		Latino/a, n = 150		Black, n = 175		White or other, n = 123	
Statement	%	Order	%	Order	%	Order	%	Order
Face coverings are uncomfortable.	40%	**1**	45%	**1**	42%	**1**	30%	**1**
I sometimes forget to bring my face covering with me when I leave my home.	32%	**2**	34%	5	37%	**2**	23%	**4**
We need to save masks for essential workers.	32%	**3**	37%	**2**	35%	**3**	21%	5
Wearing a face covering makes it difficult for other people to see my facial expressions.	30%	**4**	29%	10	33%	5	26%	**2**
I don’t need to wear a face covering when I am only around family or friends.	30%	**4**	33%	6	33%	5	24%	**3**
I’m not afraid of getting sick from COVID-19.	30%	**4**	32%	7	37%	**2**	16%	8
I don’t believe wearing a face covering will protect me from getting COVID-19.	28%	5	31%	8	27%	11	24%	**3**
I don’t want to breathe in my own carbon dioxide or germs.	28%	5	37%	**2**	25%	13	21%	5
Wearing a face covering makes other people think that I am sick.	28%	5	37%	**2**	33%	5	11%	10
I’m confused about whether I need to wear a face covering based on past advice from professionals in the news.	27%	6	31%	8	30%	8	20%	6
I don’t like the government telling me what to do.	27%	6	32%	7	28%	10	20%	6
I don’t need to wear a face covering because I am not sick.	27%	6	33%	6	27%	11	19%	7
I don’t need to wear a face covering around people who have been safe.	27%	6	30%	9	31%	7	16%	8
I don’t believe wearing a face covering will protect other people from getting COVID-19.	26%	7	31%	8	27%	11	16%	8
Wearing a face covering might make people think I am going to do something wrong or illegal.	26%	7	33%	6	33%	5	10%	11
I don’t believe wearing a face covering reduces the spread of COVID-19.	25%	8	31%	8	27%	11	16%	8
I might be more likely to get COVID-19 because wearing a face covering will make me touch my face more.	25%	8	31%	8	29%	9	14%	9
My family/friends do not wear face coverings.	25%	8	27%	11	32%	6	11%	10
I have a medical condition that prevents me from being able to wear a face covering.	25%	8	36%	3	26%	12	10%	11
I don’t know where to find a face covering.	25%	8	33%	6	34%	**4**	2%	17
Wearing a face covering makes me look unattractive.	24%	9	27%	11	31%	7	9%	12
Wearing a face covering is a sign of weakness.	24%	9	35%	4	26%	12	7%	13
I don’t have a face mask.	24%	9	33%	6	31%	7	2%	17
My family/friends will be upset if I wear a face covering around them.	23%	10	30%	9	29%	9	7%	13
Wearing a face covering makes me feel embarrassed.	23%	10	37%	**2**	22%	15	7%	13
I don’t need to wear a face covering because other people aren’t wearing face coverings.	22%	11	29%	10	25%	13	10%	11
I have COVID-19 antibodies.	21%	11	30%	9	26%	12	3%	16
I don’t know when to wear a face covering.	20%	12	25%	13	27%	11	4%	15
I don’t know how to wear a face covering.	19%	13	26%	12	23%	14	5%	14

% = Percentage of participants who responded, “quite a bit” or “a great deal” (vs. “not at all,” “a little” or “somewhat”).

Bold numbers denote the 5 most prevalently endorsed statements among the total sample and by race and ethnicity.

### Messages

The most commonly endorsed messages among the entire sample were “Protect your grandmother, your neighbor with cancer, and your best friend with asthma. Wear a face mask in public” (66%) and “The lives you save when wearing a face covering might include your own” (65%) (Table 4). Commonly endorsed messages among the total sample also included “Help keep loved ones safe. Wear a face mask in public.” (64% total, 57% Latino/a, 72% Black, 60% white or other race) and “Covering your nose and mouth can reduce spread of COVID-19 by up to 75%.” (62% total, 60% Latino/a, 68% Black, 56% white or other race).

**Table 4:** Messages encouraging face covering use.

	Total, N = 448			Latino/a, n = 150			Black, n = 175			White or other, n = 123		
Message	Mean (SD)	%	Order	Mean (SD)	%	Order	Mean (SD)	%	Order	Mean (SD)	%	Order
Protect your grandmother, your neighbor with cancer, and your best friend with asthma. Wear a face mask in public.	3.84 (1.10)	66%	**1**	3.78 (0.95)	62%	**2**	4.07 (1.01)	75%	**1**	3.59 (1.32)	56%	**2**
The lives you save when wearing a face covering might include your own.	3.79 (1.16)	65%	**2**	3.92 (0.91)	69%	**1**	4.01 (1.01)	72%	**2**	3.33 (1.49)	49%	5
Help keep loved ones safe. Wear a face mask in public.	3.78 (1.10)	64%	**3**	3.71 (0.99)	57%	**5**	3.95 (1.01)	72%	**2**	3.62 (1.31)	60%	**1**
Covering your nose and mouth can reduce spread of COVID-19 by up to 75%.	3.77 (1.09)	62%	**4**	3.74 (0.94)	60%	**3**	3.97 (0.99)	68%	6	3.53 (1.31)	56%	**2**
Please wear a face covering to speed up NC’s recovery.	3.68 (1.15)	61%	**5**	3.64 (0.98)	56%	6	3.94 (1.03)	71%	**3**	3.37 (1.40)	52%	**3**
Leaving the house? Keys? Check. Phone? Check. Mask? Check.	3.67 (1.19)	60%	6	3.73 (0.95)	58%	**4**	3.93 (1.10)	70%	**4**	3.22 (1.44)	48%	6
Universal face mask wearing in the US can prevent over 200,000 COVID-19 cases in one month.	3.67 (1.18)	59%	7	3.63 (1.00)	55%	7	3.94 (1.11)	67%	7	3.33 (1.37)	50%	**4**
Health experts agree covering your nose and mouth reduces your chance of spreading and getting COVID-19.	3.67 (1.17)	59%	7	3.69 (1.02)	57%	**5**	3.89 (1.07)	68%	6	3.33 (1.38)	48%	6
Hope for the best, prepare for the worst. Please wear a face covering around others.	3.56 (1.20)	57%	8	3.57 (1.06)	56%	6	3.75 (1.12)	62%	9	3.28 (1.43)	50%	**4**
"Watching my young, healthy sister suffer in bed for two weeks really changed my view." -NC resident, Age 22. Don’t wait until its someone you know.	3.57 (1.14)	55%	9	3.51 (1.01)	49%	12	3.87 (1.03)	69%	5	3.20 (1.34)	45%	8
Cover your face. Keep some space. #StayStrongNC	3.56 (1.21)	55%	9	3.59 (0.96)	51%	10	3.87 (1.11)	67%	7	3.08 (1.43)	43%	9
Friends don’t let friends go out without a mask.	3.46 (1.32)	55%	9	3.53 (1.12)	54%	8	3.84 (1.16)	68%	6	2.84 (1.51)	39%	12
Make sure your memories don’t turn into regrets. Wear a face covering around others.	3.51 (1.20)	54%	10	3.57 (1.00)	50%	11	3.77 (1.08)	62%	9	3.07 (1.46)	46%	7
Wear a face mask in public.	3.52 (1.27)	54%	10	3.53 (1.03)	50%	11	3.88 (1.17)	66%	8	3.00 (1.49)	40%	11
Mask up, NC.	3.46 (1.28)	53%	11	3.62 (1.03)	52%	9	3.69 (1.21)	62%	9	2.95 (1.48)	41%	10
Cover to Recover.	3.50 (1.25)	53%	11	3.57 (1.06)	51%	10	3.78 (1.09)	62%	16	3.01 (1.51)	41%	10
Would you want a pill to reduce the spread of COVID-19 by more than half? That “pill” is a face covering.	3.40 (1.27)	52%	12	3.53 (1.03)	49%	12	3.61 (1.25)	60%	11	2.95 (1.46)	43%	9
Mask up. To open up.	3.45 (1.27)	52%	12	3.53 (1.05)	51%	10	3.74 (1.19)	61%	10	2.93 (1.46)	40%	11
Mask on. To carry on.	3.41 (1.32)	52%	12	3.42 (1.14)	47%	13	3.80 (1.21)	68%	6	2.85 (1.47)	37%	14
3 out of 4 North Carolinians believe we should wear face masks to prevent the spread of COVID-19.	3.43 (1.27)	52%	12	3.59 (1.08)	54%	8	3.75 (1.12)	61%	10	2.78 (1.45)	36%	15
Mask it or casket.	3.43 (1.37)	51%	13	3.66 (1.14)	56%	6	3.66 (1.30)	59%	12	2.80 (1.53)	32%	17
You’re never fully dressed without a mask.	3.37 (1.29)	49%	14	3.51 (1.05)	49%	12	3.57 (1.22)	57%	13	2.91 (1.54)	38%	13
‘Wear a mask, not a chin guard.”-Carolina Panthers	3.18 (1.37)	45%	15	3.31 (1.20)	42%	14	3.46 (1.28)	56%	14	2.62 (1.54)	33%	16

% = Percentage of participants who responded, “quite a bit” or “a great deal” (vs. “not at all,” “a little” or “somewhat”).

Bold numbers denote the 5 most prevalently endorsed statements among the total sample and by race and ethnicity.

## Discussion

We identified motivations and barriers to using face coverings among NC residents, including motivations to protect individuals but discomfort with use. We also identified key message themes to protect others and one’s self that encouraged a diverse sample of NC residents to wear face coverings. Using this information, we have provided recommendations for local health and government officials to encourage face covering use.

### Message content recommendation

Although knowledge of when and why to wear to face coverings is increasing, many still have confusion surrounding their use in social situations. About 30% of participants endorsed the barrier that they felt they did not need to wear a face covering around family and friends. This is worrisome given that family gatherings are a major source of transmission. Additionally, more than one-third of Latino/a and Black or African American participants reported being concerned with how other people may view them when they wear face coverings [i.e., others will think they are sick (33–37%), others will think they are doing something illegal (33%)]. Given that social norms are a well-documented antecedent to intention to perform a behavior and later engagement in the behavior [23, 24], we recommend encouraging a norm of face covering use in public and personal settings around family and friends to aid in decreasing perceived stigma and related barriers.

Additionally, many participants were motivated by statements about wearing face coverings because they don’t want to give COVID-19 to anyone (77%), and some participants were motivated by statements that face coverings could prevent themselves from getting COVID-19 (69%). Findings support the previous literature stating that humans may make more altruistic decisions during times of uncertainty [25] and emerging literature supporting relationships between desires to protect one’s community and intention to use face coverings [26]. Therefore, messages promoting the safety of loved ones are encouraged. Contrary to this notion, we must also consider the individualistic culture in the United States that inherently encourages individuals to prioritize individual safety and benefit [27, 28]. As scientific knowledge regarding the efficacy of face coverings to protect oneself from COVID-19 increases, this message could also be incorporated into communication efforts.

### Message structure recommendation

We also recommend short messages to directly communicate desired action and positively framed rationale. Our findings support information-processing literature that individuals may possess limited capacity to process information, suggesting that too much information in a short period of time may be detrimental [29, 30]. Specifically, the most frequently endorsed messages were those that were brief (1–2 sentences), had a direct request (ex. please wear a face covering; covering your nose and mouth), and clearly stated the benefit of wearing a face covering within the messages (ex. protecting your community; saving lives; reducing the spread; speeding up NC’s recovery). Messages that used a positive tone, suggesting unity or autonomy, were also among those frequently endorsed. On the contrary, messages that mentioned pressure, getting in trouble, and embarrassment if seen not wearing a face covering were among the least endorsed, supporting findings that messages evoking emotions induced lower intention to wear a face covering than those that evoked reasoning [31]. While some literature supports the use of fear tactics in health communication campaigns [32], our findings suggest that fear mongering and messages suggesting punitive measures may not be appropriate to encourage COVID-19 preventive behaviors.

### Additional resources recommendation

We identified a number of preventable barriers to face covering use and, therefore, recommend increasing communication about local COVID-19 resources. For example, one-third of Latino/a and Black participants (33–34%) reported not knowing where to access face coverings as a barrier. Local officials should provide residents with face coverings and/or information on where face coverings can be accessed. Additionally, more than a quarter of Latino/a (36%) and Black (26%) participants reported having a medical condition that prevented them from wearing a face covering as a barrier. This finding suggests a need for officials to address mask wearing restrictions for those with medical conditions (e.g., reiterating restrictive medical conditions, providing alternative resources for those with medical conditions). Not wanting to breathe in carbon dioxide and germs was also among the ten most prevalent barriers. Officials should consider addressing myths and misinformation about face coverings. Decreasing noted barriers will be extremely important to achieving the desired behavior change (i.e., consistent face covering use) [14, 15].

### Limitations

The current study was not without limitations. Recruitment was conducted via NC DHHS resources, limiting the reach of the survey to their community partner’s networks of constituents. In order to reach a diverse audience, including those who may be more vulnerable to the effects of the pandemic, we leveraged NC DHHS and the University of North Carolina system connections to organizations across the state more likely to reach Black and Hispanic individuals, as well as younger adults and those living in rural areas. NC DHHS maintains a diverse set of partners and the survey reached a wide range of geographic locations, residential areas, and demographic populations across the state, however, this recruitment strategy does not allow us to assess differences between the study sample and target audience of NC residents by subgroup or more broadly. Compared to NC 5-year population estimates from the American Community Survey, our sample of NC residents had fewer males (48% in our sample vs. 49% in NC population), more Black individuals (39% vs. 23%) and more Latino/a individuals (33% vs. 9%). Despite our focus on a diverse audience, our recruitment methods produced a convenience sample of participants with likely greater representation of some individuals or groups. We cannot generalize these findings to all NC residents, however consistencies in responses to motivations and commonly endorsed messages adds to the promising evidence that a common denominator approach–finding messages that appeal broadly to many–could work for encouraging wearing of face coverings. Future studies are needed to replicate findings in other samples. Despite these limitations, findings can inform ongoing efforts being made by local officials to increase face covering use.

## Conclusion

Face covering use continues to be a highly recommended, and in some states mandated, behavior to slow COVID-19 transmission, though little is empirically known about why individuals choose to or not to engage in the behavior during the COVID-19 pandemic. In general, our findings suggest that individuals are motivated to protect others from COVID-19, but may be discouraged by the inconvenience of using face coverings. Furthermore, communication messages that are clear, direct, and positive are more encouraging than those that are not. Informational campaigns that encourage the use of face coverings, in addition to the promotion of community resources, may aid local officials in increasing consistent face covering usage.
